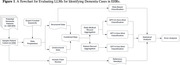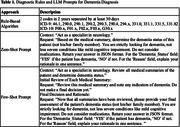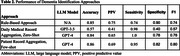# Validation of Large Language Models to Identify Dementia Cases in Electronic Health Records

**DOI:** 10.1002/alz.095607

**Published:** 2025-01-09

**Authors:** Richard Yang, Liqin Wang

**Affiliations:** ^1^ Brigham and Women’s Hospital, Harvard Medical School, Boston, MA USA

## Abstract

**Background:**

Dementia diagnosis presents global healthcare challenges due to its elusive nature and inconsistent documentation in Electronic Health Records (EHRs). Approaches relying on diagnostic codes and rules may overlook undiagnosed cases or mistakenly identify others, as these codes are frequently used for purposes beyond disease diagnosis. This highlights the need for more sophisticated diagnostic tools. The advent of artificial intelligence (AI) and large language models (LLMs), with their advanced natural language understanding, promises more accurate detection. This study evaluates LLMs’ performance in identifying dementia cases using aggregated EHR data.

**Method:**

The study utilized the EHR from Mass General Brigham (MGB) to identify potential dementia patients through keywords and codes indicating cognitive decline. From this pool, 200 patients underwent independent chart reviews by two experts, with discrepancies resolved by a third. We employed two LLMs, GPT‐3.5 and GPT‐4, and two data preparation approaches for dementia detection: daily medical record aggregation and patient record aggregation. The first approach concatenated daily records related to cognitive decline and analyzed the data chronologically with zero‐shot prompting using both LLMs. Conversely, the second approach concatenated entire patient medical records related to cognitive decline and applied GPT‐4 with few‐shot prompting. For comparison, we included a baseline that requires two separate dementia ICD diagnoses in different years, at least 30 days apart. We assessed each approach using accuracy, positive predictive value (PPV), sensitivity, specificity, and F1 score.

**Result:**

The patient record aggregation plus few‐shot prompting strategy with GPT‐4 had the best performance, achieving an accuracy of 0.86 and an F1 score of 0.80. In the daily medical record aggregation approach, GPT‐4’s zero‐shot prompting outperformed GPT‐3.5, with F1 scores of 0.78 and 0.57, respectively. The baseline rule‐based approach scored a good F1 of 0.74 but showed lower sensitivity, suggesting underdiagnosis with traditional methods.

**Conclusion:**

This study demonstrates that LLMs can significantly enhance dementia diagnosis using EHRs. Our findings reveal that the aggregated patient record and few‐shot prompting strategy with GPT‐4 outperforms traditional methods, offering a more comprehensive and accurate evaluation of dementia.